# Simultaneous Optimization for Ultrasound-Assisted Extraction and Antioxidant Activity of Flavonoids from *Sophora flavescens* Using Response Surface Methodology

**DOI:** 10.3390/molecules24010112

**Published:** 2018-12-29

**Authors:** Jing Zhou, Lincheng Zhang, Qinping Li, Weifeng Jin, Weiyan Chen, Jin Han, Yuyan Zhang

**Affiliations:** 1 College of Life Science, Zhejiang Chinese Medical University, 548 Binwen Road, Binjiang District, Hangzhou 310053, Zhejiang, China; zhoujing00727@163.com (J.Z.); 18758132027@163.com (Q.L.); 2 Second Clinical Medical College, Zhejiang Chinese Medical University, 548 Binwen Road, Binjiang District, Hangzhou 310053, Zhejiang, China; ZLC1998327@163.com; 3 College of Pharmacy, Zhejiang Chinese Medical University, 548 Binwen Road, Binjiang District, Hangzhou 310053, Zhejiang, China; jin_weifeng@126.com; 4 College of Basic medical, Zhejiang Chinese Medical University, 548 Binwen Road, Binjiang District, Hangzhou 310053, Zhejiang, China; chengweiyan18@sina.com (W.C.); hanjin20181127@sina.com (J.H.)

**Keywords:** *Sophora flavescens*, flavonoids, ultrasound-assisted extraction, antioxidant activity, response surface methodology

## Abstract

The ultrasonic-assisted extraction process and antioxidant activity of flavonoids from *Sophora flavescens* were investigated in this study. In order to optimize the extraction of flavonoids from *Sophora flavescens*, the influence of extraction time, methanol concentration, ultrasonic temperature, and solvent-to-material ratio was analyzed. Results showed that the extraction yields reached a maximum with the extraction time of 30 min, methanol concentration of 80%, temperature of 80 °C, and solvent-to-material ratio of 26 mL/g. The flavonoids were determined by HPLC, and the mean yields of trifolirhizin, formononetin, isoxanthohumol, maackiain, and kurarinone under the optimal conditions were 2.570, 0.213, 0.534, 0.797, and 3.091 mg/g, respectively. The evaluation of vitro antioxidant activity exhibited *Sophora flavescens* flavonoids had a strong 1,1-diphenyl-2-picrylhydrazyl (DPPH) and hydroxyl radical-scavenging ability with IC50 of 0.984 and 1.084 mg/g, respectively. These results indicate that ultrasonic-assisted extraction is an efficient approach for the selective extraction of flavonoids, and response surface methodology further optimized the extraction.

## 1. Introduction

*Sophora flavescens*, the dry root of *Sophora flavescens* Ait., is a widely used traditional Chinese medicine that has the functions of clearing heat and diuresis, expelling dampness and anthelmintic [1]. The chemical constituents in *Sophora flavescens* are mainly alkaloids and flavonoids. Previous studies and clinical research mostly focused on the alkaloids [2,3,4,5]. However, recent attention has been attracted by isopentenyl flavonoids contained in *Sophora flavescens* [6,7]. Isopentenyl flavonoids from *Sophora flavescens* have a variety of biological and chemical activities, and are the reason for the comprehensive evaluation of its quality [1]. Furthermore, it has been well demonstrated that isopentenyl flavonoids have extensive pharmacological activity and clinical applications such as bacteriostasis, antitumor, anti-inflammation, anti-arthritic, and in the regulation of dyslipidemia [8,9,10]. Flavonoids are compounds with a basic carbon framework of C6-C3-C6, mostly present in the form of glycosides or carbohydrates in plants. It usually poorly water soluble, thus normally organic solvents are required to extract flavonoids from *Sophora flavescens*.

Diverse techniques such as conventional heat reflux extraction, microwave-assisted extraction, and supercritical fluid extraction has been widely used for the extraction of flavonoids compounds [11]. Compared with other techniques, ultrasonic-assisted extraction is commonly used because of its high efficiency, time-saving simplified manipulation, and because it is environmental-friendly [12].

Ultrasonic-assisted extraction (UAE) is an efficient and environmentally friendly approach and has been widely used for plants extraction [13,14]. Exactly, the propagation of ultrasonic mechanical thermal vibration contributes to the dissolution and diffusion of the active constituents of the plant. The cavitation releases energy and generates instantaneous high temperature and pressure to cause the cell wall to be cracked, which impels the contents directly dissolved and fully contacted with the solvent [14,15,16]. As a result, the yields of the extracts increase. Response surface methodology (RSM) is a feasible mathematical and statistical model to achieve the efficient analysis of experimental data and optimization of extraction parameters [17,18].

The extraction process is affected by various factors, such as extraction time, temperature, pH, ultrasonic power, solvent, and others. It is already well established that extraction time, methanol concentration, temperature, and solvent-to-material ratio play an important role in the yield of flavonoids [1,19]. Traditional Chinese medicine is a natural antioxidant with great potential and safety. In this study, UAE was integrated with RSM, as well as HPLC, to optimize combinations of flavonoids including trifolirhizin, formononetin, isoxanthohumol, maackiain, and kurarinone. The extraction process is based on the single factor experiment and response surface methodology. In addition, the antioxidant activity of *Sophora flavescens* extracts is evaluated by determining the 1,1-diphenyl-2-picrylhydrazyl (DPPH) and hydroxyl scavenging activity compared with ascorbic acid as an antioxidant standard [20,21,22]. It may provide a theoretical basis for further development and utilization of *Sophora flavescens*.

## 2. Results

### 2.1. Single-Factor Experiment of Flavonoids Extraction

#### 2.1.1. Effect of Ultrasonic Time

**Figure 1:**
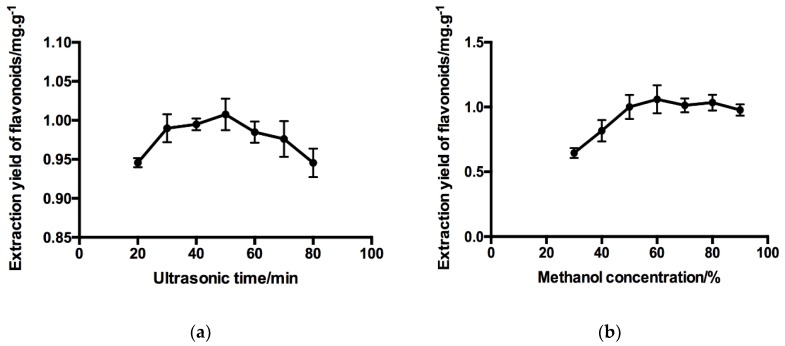

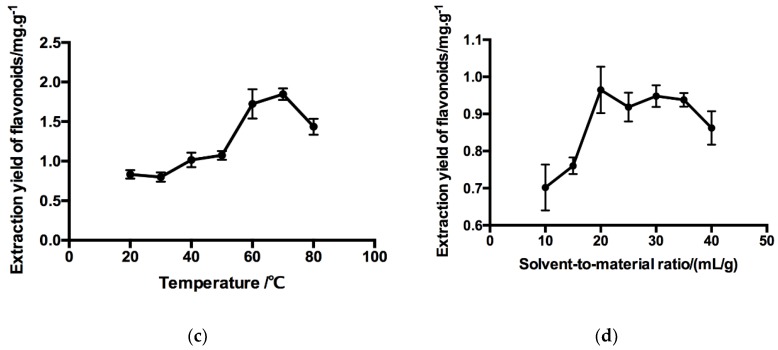
Effect of dependent variables on the extraction yield of flavonoids (**a**) extraction temperature, (**b**) methanol concentration, (**c**) temperature, and (**d**) solvent-to-material ratio. Data represent mean ± SD of three independent experiments.

The effect of ultrasonic time on total flavonoids extraction yield is shown in Figure 1a. The extraction was continued for 20, 30, 40, 50, 60, 70, and 80 min, respectively. The extraction temperature was fixed to 50 °C and ultrasonic power fixed to 120 W. Sixty per cent methanol was selected as solvent, and the liquid-to-material ratio was fixed to 20:1 mL/g, all other conditions being equal. With the prolongation of ultrasonic extraction time, the yield of flavonoids increased. After the extraction time lasted for 50 min, the yield of flavonoids reached its maximum, followed by a slow declining trend. It is speculated that prolonged ultrasonic time may degrade flavonoids or accompany the dissolution of other impurities. For comprehensive consideration, ultrasonic time of 30–50 min was used in the RSM experiment.

#### 2.1.2. Effect of Solvent

**Figure 2:**
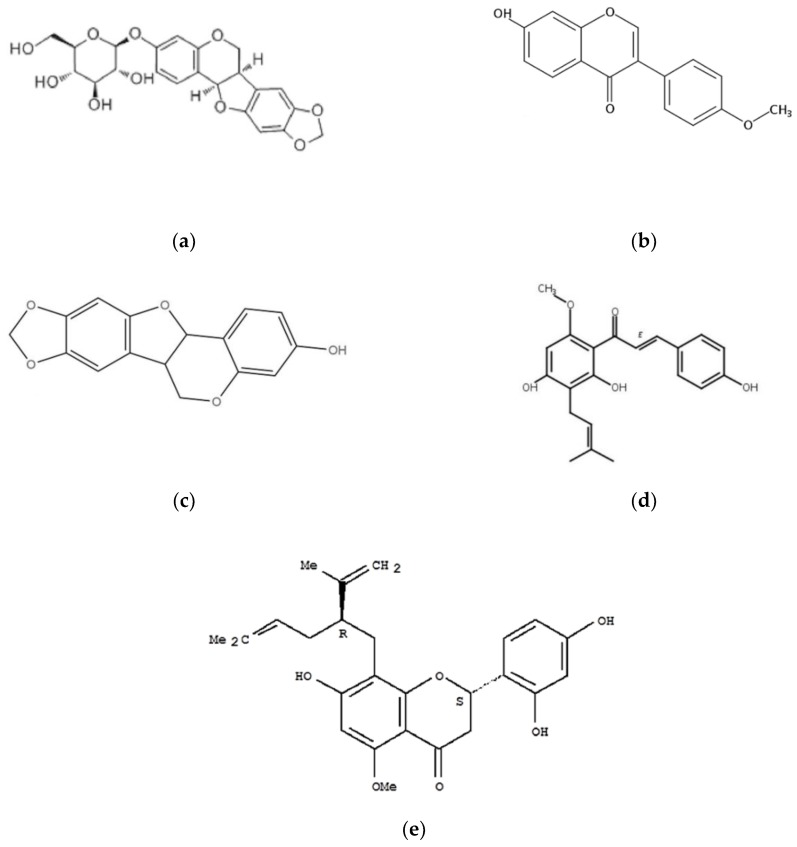
Structure of the five flavonoids: (**a**) trifolirhizin; (**b**) formononetin; (**c**) isoxanthohumol; (**d**) maackiain; and (**e**) kurarinone.

In general, solvent is considered an important parameter for UAE: it may affect the solubility of the target ingredients [20]. Generally, ethyl acetate, ethanol, and methanol are the most hackneyed solvents for extraction, but different solvents are suitable for different flavonoids compounds. According to the theory of similar dissolve mutually and previous research, the extraction using methanol has a higher yield and better solubility of flavonoids [21]. Based on the structure of the five flavonoids compounds (Figure 2), it is speculated that higher methanol ratio is propitious to the dissolution of these substances.

In this single-factor experiment, we investigated the effect of different methanol concentration (30–90% (*v*/*v*)) on flavonoid extraction. As shown in Figure 1b, the yield of flavonoids kept steady increasing when the concentration of methanol ranged from 30% to 60%. When methanol concentration exceeded 60%, the yield of flavonoids decreased weakly. It could be caused by the increasing dissolution of other alcohol-soluble substances. Therefore, the variable range of methanol concentration used in the RSM experiments was selected as 60–80%.

#### 2.1.3. Effect of Temperature

As the results shows in Figure 1c, a significant increase in flavonoid yield was observed by increasing the extraction temperature from 20 °C to 70 °C because higher extraction temperatures accelerate molecular motion, penetration, dissolution, and diffusion in favor of releasing the flavonoids. The peak yield (1.85 ± 0.072 mg/g) was reached at an extraction temperature of 70 °C, and the yield decreased with a further increase in the extraction temperature. The yield of flavonoids in 80 °C was significantly higher than yield in 50 °C (*p* < 0.05), thus the variable temperature range used in the RSM experiments was selected as 60–80 °C.

#### 2.1.4. Effect of Liquid-to-Material Ratio

UAE procedure is executed at different liquid-to-material ratios while fixing the other extraction parameters as follows, extraction temperature, 50 °C; methanol concentration, 60%; and extraction time, 30 min. As shown in Figure 1d, extraction yield was affected by the liquid-to-material ratio. With an increased ratio from 10:1 mL/g to 20:1 mL/g, the flavonoids yield increased and reached the maximum value (0.965 ± 0.062 mg/g) at 20:1 mL/g. When the liquid-to-material ratio was further increased, an induction in flavonoids was observed. It indicates that a liquid-to-material ratio that is too low or too high may not favor the movement of the plant cells and the flavonoids to the solvents under ultrasonic treatment [15]. No significant difference was observed between an extraction yield of 25:1 mL/g and 35:1 mL/g (*p* > 0.05). Therefore, the variable range of liquid-to-material ratio used in the RSM experiments was selected as 20:1–30:1 mL/g because solvent consumption can increase production cost.

### 2.2. Model Fitting

**Figure 3:**
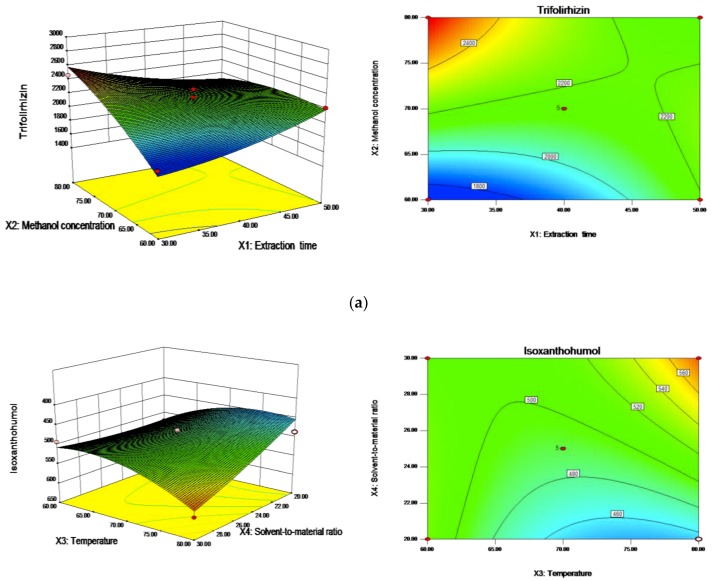

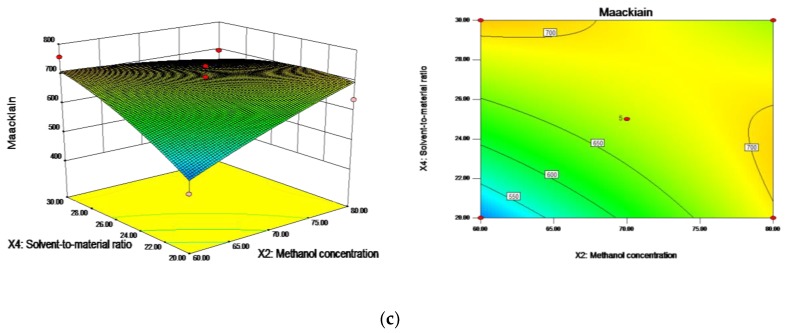
Response surfaces and contours plots of flavonoids extracted from *Sophora flavescens*: (**a**) extraction time (X_1_) and methanol concentration (X_2_); (**b**) extraction temperature (X_3_) and solvent-to-material ratio (X_4_); and (**c**) methanol concentration (X_2_) and solvent-to-material ratio (X_4_).

The BBD in the optimization experiment consisted of four factors, three levels, and five center point runs that were carried out in triplicate. The experimental conditions and results of 29 runs are presented in Table 1. The results of the analysis of variance (ANOVA) are shown in Table 1. *p*-values were used to check the significance of each coefficient. Specifically, values of “probability (*p*) > F” of less than 0.05 and 0.01 indicate that the model terms are significant and highly significant, respectively, and values greater than 0.05 indicate that the model terms are not significant [23,24]. F values revealed that the model was statistically significant (*p* < 0.05) for five flavonoids of trifolirhizin, formononetin, Isoxanthohumol, maackiain, and kurarinone. The lack of fit of each model was not significant (*p* > 0.05), indicating that the developed model adequately explains the relationship between the independent variables and responses. The values of determination coefficients (R^2^) and adjusted determination coefficients (Adj. R^2^) are shown in Table 1. These indicated the response was sufficiently explained by the model. The generated response surface 3D graphs corresponding to each response showed the interactive effects of the variables, if any (Figure 3a–c).

### 2.3. Effect of the Variables on the Extraction Yield of Flavonoids

#### 2.3.1. Trifolirhizin

In Table 1, the ANOVA results showed significant linear (X_2_ and X_4_) and interactive (X_1_X_2_) effects on trifolirhizin. Based on the regression coefficient (β) values, linear (X_2_) revealed a major effect, which was followed by X_4_ and X_1_X_2_. The extraction yield value of trifolirhizin can be expressed as the following second order polynomial equation.

Y_1_ = 2156.02 + 7.09X_1_ + 203.12X_2_ − 30.26X_3_ + 107.87X_4_ − 238.19X_1_X_2_ + 38.95X_1_X_3_ + 61.92X_1_X_4_ + 32.12X_2_X_3_ + 14.6X_2_X_4_ − 103.3X_3_X_4_ + 75.39X_1_^2^ − 86.69X_2_^2^ + 90.78X_3_^2^ + 26.23X_4_^2^ (1)

#### 2.3.2. Formononetin

The solvent-to-materials ratio exerted a linear and quadratic effect on the yields of formononetin. The extraction yield value of trifolirhizin can be expressed by the following second order polynomial equation. The relationship between formononetin extraction yield and variables can be described as a second order polynomial equation as follows

Y_2_ = 191.81 − 1.37X_1_ + 3.89X_2_ − 7.3X_3_ + 16.56X_4_ − 12.3X_1_X_2_ − 1.05X_1_X_3_ + 18.71X_1_X_4_ + 13.57X_2_X_3_ − 16.75X_2_X_4_ − 8.68X_3_X_4_ + 9.63X_1_^2^ − 4.26X_2_^2^ + 16.32X_3_^2^ − 23.93X_4_^2^ (2)

#### 2.3.3. Isoxanthohumol

As show in Table 1, the solvent-to-materials ratio (X_3_) has remarkable significance (*p* < 0.01) on isoxanthohumol, and the quadratic effect of temperature (X_3_^2^) and the interaction (X_3_X_4_) also make a difference on the extraction of isoxanthohumol. The quadratic equation obtained in terms of actual factors is described as follows

Y_3_ = 490.11 + 1.97X_1_ + 4.9X_2_ + 2.44X_3_ + 28.77X_4_ − 15.68X_1_X_2_ − 12.42X_1_X_3_ + 1.01X_1_X_4_ + 19.25X_2_X_3_ − 17.01X_2_X4 + 34.37X_3_X_4_ + 2.36X_1_^2^ − 16.77X_2_^2^ + 31.07X_3_^2^ − 6.43X_4_^2^ (3)

#### 2.3.4. Maackiain

The linear effects of the methanol concentration (X_2_) (*p* < 0.05), temperature (X_3_) (*p* < 0.05), and solvent-to-material ratio (X_4_) (*p* < 0.01), as well as the interaction of X_2_X_4_ showed significant effects on maackiain. The quadratic equation is described as follows

Y_4_ = 675.45 − 14.06X_1_ + 37.05X_2_ − 35.14X_3_ + 44.03X_4_ − 45.85X_1_X_2_ − 31.24X_1_X_3_ + 24.88X_1_X_3_ + 13.43X_2_X_3_ − 60.72X_2_X_4_ + 37.21X_3_X_4_ − 5.72X_1_^2^ − 9.61X_2_^2^ − 7.62X_3_^2^ − 24.05X_4_^2^ (4)

#### 2.3.5. Kurarinone

Kurarinone is primarily affected by methanol concentration (X_2_) and solvent-to-liquid ratio (X_4_) (*p* < 0.05). The quadratic effect of temperature(X_3_) also works in the extraction of kurarinone. The model of kurarinone can be represented as follows

Y_5_ = 2632.55 + 38.28X_1_ + 116.87X_2_ + 68.21X_3_ + 113.83X_4_ + 72.16X_1_X_2_ − 61.74X_1_X_3_ − 243.38X_1_X_4_ 70X_2_X_3_ − 33.67X_2_X_4_ + 70.7X_3_X_4_ + 194.62X_1_^2^ − 83.15X_2_^2^ + 51.19X_3_^2^ + 61.22X_4_^2^ (5)

### 2.4. Optimal Processing Conditions and Model Verification

Based on the regression analysis and 3D surface plots of the independent variables, the optimum conditions for the maximum flavonoids (trifolirhizin, formononetin, isoxanthohumol, maackiain, and kurarinone) using comprehensive evaluation value were obtained with an extraction time 30 min, methanol concentration of 79.98% (*v*/*v*), extraction temperature of 80 °C, and solvent to material ratio of 26.25 mL/g. For the convenience of experiments, parameters were modified slightly in the verification experiment as follows, extraction time 30 min, methanol concentration of 79.98% (*v*/*v*), extraction temperature of 80 °C, and solvent-to-material ratio of 26.25 mL/g. All experiments under the optimized conditions were performed in quintuplicate, and the results are displayed in Table 2.

### 2.5. Antioxidant Activity In Vitro

#### 2.5.1. DPPH Radical Scavenging Activity

**Figure 4:**
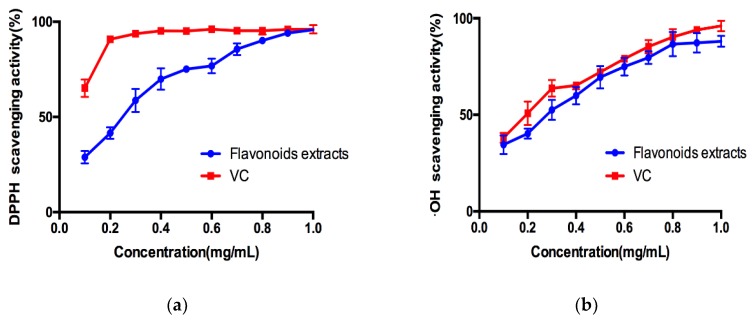
Antioxidant activity of *Sophora flavescens* extracts. (**a**) 1,1-diphenyl-2-picrylhydrazyl (DPPH) radical scavenging activity. (**b**) Hydroxyl radical scavenging activity. Data represent means ± SD of three independent experiments.

DPPH scavenging ability is a common used method to appraise antioxidant activity of natural compounds. The antioxidant activity of flavonoids from *Sophora flavescens* was evaluated with DPPH-scavenging assay, in comparison with known antioxidant, ascorbic acid (VC). As shown in Figure 4a, the DPPH radical scavenging activity of flavonoids presented notable DPPH radical-scavenging activity, and the capacity were increased with increasing concentrations. Obviously, the scavenging effects of extractions were weaker than that of VC in same concentration. However, when the concentrations of flavonoids increased to 1.00 mg/mL, the antioxidant activity (95.83 ± 0.27%) of flavonoids was approximate to that of VC (96.04 ± 2.20%) (Figure 3). These results indicated that *Sophora flavescens* extracts had a strong DPPH radical scavenging activity, with an IC50 value of 0.984 mg/g.

#### 2.5.2. Hydroxyl Radical Scavenging Activity

The hydroxyl radical, which is well known as one of the most reactive free radicals, can trigger free radical chain reactions and attack biological molecules to induce severe damage. As shown in Figure 4b, *Sophora flavescens* extracts exhibited a lower capacity of hydroxyl radical scavenging activity than that of VC. It displayed a concentration-dependent antioxidant activity with a similar trend to VC, and an IC50 value of 1.084 mg/g. These results proved that flavonoids obtained from *Sophora flavescens* possess the ability to be good antioxidants.

### 2.6. HPLC Analysis of Flavonoids

**Figure 5:**
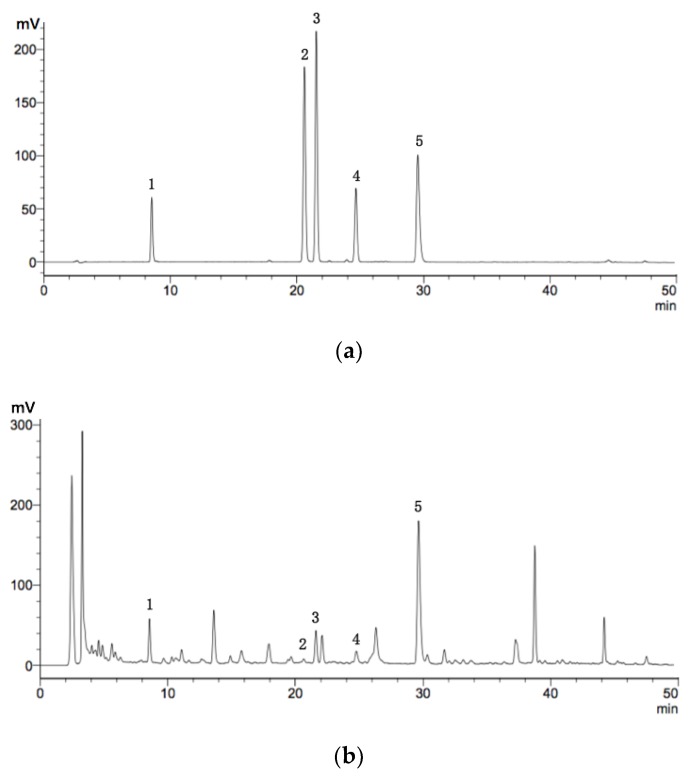
HPLC chromatogram of mixed reference (**a**) and extraction samples (**b**). 1: trifolirhizin; 2: formononetin; 3: isoxanthohumol; 4: maackiain; 5: kurarinone.

Five flavonoids of *Sophora flavescens* were identified by HPLC and detected at wavelengths of 295 nm (Figure 5). The content of trifolirhizin, formononetin, isoxanthohumol, maackiain, and kurarinone was determined by a corresponding conversion of the peak area. Pharmacological and nutritional studies have found that these five compounds exhibited various activities such as antioxidant, bacteriostasis, antitumor, and anti-inflammatory effects [8,25,26,27]. The combined effects of these flavonoids and other compounds may affect antioxidant activities by scavenging free radicals and inhibiting lipid peroxidation [28,29,30]. Therefore, these five constituents in the extracts may be partly responsible for the antioxidant activity observed in flavonoids obtained by UAE.

## 3. Materials and Methods

### 3.1. Plant Materials

*Sophora flavescens* was purchased from the Traditional Chinese Herbal Medicine Co., Ltd. (Hangzhou, China) of Zhejiang Chinese Medical University and identified by Xilin Chen, the professor of Zhejiang Chinese Medical University. It was crushed using a mill (TAISITE, Tianjin, China) and sifted through a 50-mesh seive, stored in a dry place before experimental study.

### 3.2. Chemicals and Reagents

Methanol (HPLC grade) was obtained from Tianjin Siyou (Tianjin, China) and acetonitrile (HPLC grade) was purchased from Tedia (Fairfield, OH, USA). Trifolirhizin, formononetin, Isoxanthohumol, maackiain, kurarinone (HPLC > 98%), and 1,1-diphenyl-2-trinitrophenylhydrazine (DPPH) were purchased from Shanghai Yuanye Biological Technology Co., Ltd. (Shanghai, China). Ascorbic acid was purchased from Drmaolab (Xilong Scientific, Shanghai, China). All other reagents used were analytically pure.

### 3.3. Ultrasound-Assisted Extraction (UAE) of Flavonoids

Dry *Sophora flavescens* powder (0.5 g) was placed into a 50 mL stuffed conical flask and then the different solvents were added. The container was capped, and the system was started-up. Ultrasonic processing was executed in the thermostatic ultrasonic cleaner (ZL3-120, Shanghai Zuole instruments Co., Ltd., Shanghai, China), equipped with a fixed ultrasound power (120W) and digital controlled low-frequency sonotrode (40 kHz). The instrument also equipped with timing device and temperature displayed digital controller to control the medium temperature. The extraction temperature and time were set at different degrees according to different conditions. After extraction, the extraction vessels were left for several minutes to cool down to room temperature. Then each extract was filtered and methanol was added to original volume [18].

### 3.4. Determination of Flavonoids

The content of flavonoids was determined by spectrophotometry using the aluminum nitrate colorimetric method with some modifications [17,27]. Briefly, 1 mL extracted solution containing flavonoids was added to a 10 mL volumetric flask. Four milliliters of 60% (*v*/*v*) methanol and 0.16 mL of 5% (*w*/*w*) NaNO_2_ were mixed for 5 min, and then 0.1 mL of 10% Al(NO_3_)_3_ (*w*/*w*) was added and mixed, 6 min later, 1 mL of 4% (*w*/*w*) NaOH was added. After 10 min, the absorbance of the solution at 510 nm was measured with a double beam UV/Vis spectrophotometer (TU-1900, Pgeneral, Beijing, China) against the same mixture without the sample as a blank. The calibration curve was prepared by preparing rutin solutions at concentrations from 20 to 100 μg/mL in methanol. The concentration of total flavonoids in extract was expressed as mg of rutin equivalents per gram dry weight of extract. The calibration curve was determined to y = 5.895x + 0.0733 and R^2^ = 0.993, where y is absorbance value of sample and x is sample concentration (10–100 μg/mL).

### 3.5. Selection of Experimental Factors and Levels

The ultrasonic time (20, 30, 40, 50, 60, 70, 80 min), methanol concentration (40%, 50%, 60%, 70%, 80%), extraction temperature (20, 30, 40, 50, 60, 70, 80 °C), and the ratio of solvent to material (10:1, 15:1, 20:1, 25:1, 30:1, 35:1, 40:1 mL/g) were investigated for the effect on the extraction of flavonoids from *Sophora flavescens* according to the method described in Section 2.3.

### 3.6. BBD Experimental Design

A four-factor-three-level Box–Behnken Design (BBD) was used [20]. The effect of independent variables (extraction time, X1; extraction temperature, X2; methanol concentration, X3; and solvent-to-material ratio, X4) on the UAE of total flavonoids from *Sophora flavescens* was estimated. On the basis of preliminary experiments, all variables were fixed at 3 levels, denoted by −1, 0, and 1, the experimental designs of the coded (x) and actual (X) levels of variables are shown in Table 3. The response values (Y) of trifolirhizin (Y_1_), formononetin (Y_2_), isoxanthohumol (Y_3_), maackiain (Y_4_), kurarinone (Y_5_) in each trial were analyzed using Design Expert 8.0.6 and fitted to a second-order polynomial regression model as follows

(6) $$Y=\beta_{0}+\overset{4}{\underset{i=1}{\sum }}\beta_{i}X_{i}+\overset{3}{\underset{i=1}{\sum }}\overset{4}{\underset{j=i+1}{\sum }}\beta_{ij}X_{i}X_{j}+\overset{4}{\underset{i=1}{\sum }}\beta_{ii}X_{i}^{2}$$

where β_0_, β*_i_*, β_ii_, and β_ij_ are constant regression coefficients of the model, while Xi and Xj are the independent variables.

In this experiment, five flavonoids of *Sophora flavescens* were determined. In order to comprehensively evaluate the extraction yields, each component was weighted by entropy weight method [31]. The weight coefficients of trifolirhizin, formononetin, isoxanthohumol, maackiain, and kurarinone were 0.187, 0.321, 0.112, 0.206, and 0.174, respectively. The comprehensive evaluation value(Y_0_) equation is as follows

Y_0_ = 0.187Y_1_ + 0.321Y_2_ +0.112Y_3_ + 0.206Y_4_ + 0.174Y_5_ (7)

The comprehensive evaluation values are shown in Table 3.

### 3.7. High Performance Liquid Chromatography (HPLC) analysis

The flavonoids were quantified by HPLC instrument (Shimadzu LC-20A) coupled with UV detection (HPLC-UV) and equipped with a reversed-phase ZORBAX SB-C18 column (4.6 mm × 250 mm, 5 µm, Agilent Technologies Inc., Santa Clara, CA, USA). The column temperature was set at 30 °C and detection wavelength was set at 295 nm. The mobile phase consisted of acetonitrile (A) and 0.2% formic acid (B) with flow rate of 1.0 mL/min. A gradient program was conducted: 0–30 min (30–50% acetonitrile); 30–40 min (50–70% acetonitrile); and 40–50 min (70% acetonitrile). The elution was employed with 20 μL of injection sample and a 10 min re-equilibration time was used between HPLC runs.

### 3.8. Determination of Antioxidant Activities

#### 3.8.1. Assay of DPPH Radical Scavenging Activity

The scavenging effect of the flavonoids extracted by UAE on DPPH radical was based on the procedure described by previous studies with some modifications [32]. Briefly, 2.0 mL of 0.2 mM DPPH in anhydrous ethanol was added to 2.0 mL of sample at different concentrations (0.1–1.0 mg/mL). The mixture was shaken and incubated for 30 min at room temperature in dark, and the absorbance of the resulting solution was measured at 517 nm. Ascorbic acid was used as the control substance. The scavenging percentage was calculated according to the following equation.

(8) $$\mathrm{Scavenge}\;\mathrm{percentage}\%=\left(1-\frac{A_{i}-A_{0}}{A_{j}}\;\right)\times 100$$

where, A*_i_* was the absorbance of 2.0 mL sample + 2.0 mL DPPH; A_0_ was the absorbance of 2.0 mL sample + 2.0 mL anhydrous ethanol; and A*_j_* was the absorbance of 2.0 mL DPPH + 2.0 mL anhydrous ethanol.

#### 3.8.2. Assay of Hydroxyl Radical Scavenging Activity

Hydroxyl radical scavenging activity was determined according to the literature with some modifications [33]. Two milliliter sample solutions of different concentrations (0.1–1.0 mg/mL)—1 mL of salicylic acid-ethanol solution (9.0 mM), 1 mL of FeSO_4_ solution (9.0 mM), and 3.0 mL of distilled water—were mixed in a tube. The reaction was initiated by the addition of 1.0 mL H_2_O_2_ (8.8 mM) to the mixture above, and the absorbance at 510 nm was measured. The hydroxyl radical scavenging activity was calculated as follows

(9) $$\mathrm{Scavenge}\;\mathrm{percentage}\%=\left(1-\frac{A_{i}-A_{0}}{A_{j}}\;\right)\times 100$$

where, A_i_ is the absorbance of the sample; A_0_ is the absorbance of the group with deionized water instead of sample; and A_j_ is the absorbance of water instead of H_2_O_2_.

### 3.9. Statistical Analysis

The results are expressed as mean ±SD (standard deviation). Design-Expert 8.0.6 (Stat-Ease Inc., Minneapolis, MN, USA) was used to calculate the coefficients of the quadratic polynomial model and the optimization. *p*-values of less than 0.05 were considered to be statistically significant.

## 4. Conclusions

RSM is a comprehensive mathematical and statistical method that has been successfully applied for optimize multifaceted processes and evaluate the influence of multiple variables and their interactions. Experimental results indicated that both methanol concentration and solvent-to-material ratio had highly significant effects on the dependent variables. Furthermore, temperature also has an influence on the yields of maackiain, while the extraction time has little correlation with the response values. The optimum extraction conditions are as follows, extraction time, 30 min; methanol concentration, 80%; extraction temperature, 80 °C; and solvent-to-material ratio, 26:1 mL/g. Under these conditions, the verification experimental results of five flavonoids in *Sophora flavescens* detected by HPLC agreed with the predicted values. It can be said that UAE is an effective and practical method for simultaneously extracting five flavonoids from *Sophora flavescens*. Thus, this optimized extraction method could be conducive for the extraction and pharmaceutical analysis of flavonoids from *Sophora flavescens*.

## Figures and Tables

**Table 1:** Regression coefficient, standard error, and analysis of variance (ANOVA) analysis of flavonoid extraction.

Factor	Trifolirhizin	Formononetin	Isoxanthohumol	Maackiain	Kurarinone
F Value (Model)	2.68 *	3 *	2.9 *	2.76 *	2.63 *
Intercept	2156.02	191.81	490.11	675.45	2632.55
X_1_ (Time)	7.09	−1.37	1.97	−14.06	38.28
X_2_ (Methanol concentration)	203.12 **	3.89	4.90	37.05 *	116.87 *
X_3_ (Temperature)	−30.26	−7.30	2.44	−35.14 *	68.12
X_4_ (Solvent-to-sample ratio)	107.87 *	16.56 *	28.77 **	44.03 **	113.83 *
X_1_X_2_	−238.19 *	−12.30	−15.68	−45.85	72.16
X_1_X_3_	38.95	−1.05	−12.42	−31.24	−61.74
X_1_X_4_	61.92	18.71	1.01	24.88	−243.38
X_2_X_3_	32.12	13.57	19.25	13.43	70.00
X_2_X_4_	14.60	−16.75	−17.01	−60.72 *	−33.67
X_3_X_4_	−103.30	−8.68	34.37 *	37.21	70.70
X_1_^2^	75.39	9.63	2.36	−5.72	194.62
X_2_^2^	−86.69	−4.26	−16.77	−9.61	−83.15
X_3_^2^	90.78	16.32	31.07 *	−7.62	51.19
X_4_^2^	26.23	−23.93 *	−6.43	−24.05	61.22
R^2^	0.728	0.7501	0.7434	0.7339	0.7244
Adj. R^2^	0.456	0.5001	0.4679	0.4868	0.4488
F Value (Lack of Fit)	1.44	1.69	3.71	1.28	2.11

Note: * *p* < 0.05 significant, ** *p* < 0.01 highly significant.

**Table 2:** Experimental data of the verification of predicted value at optimal extraction conditions.

Varication Experiment	Trifolirhizin (mg/g)	Formononetin (mg/g)	Isoxanthohumol (mg/g)	Maackiain (mg/g)	Kurarinone (mg/g)	Comprehensive Evaluation Value	Predicted Value	Relative Error/%
1	2.696	0.225	0.561	0.870	3.004	1.341	1.345	2.55
2	2.520	0.204	0.523	0.769	3.022	1.279		
3	2.445	0.218	0.531	0.812	3.230	1.316		
4	2.555	0.207	0.524	0.765	2.976	1.278		
5	2.633	0.208	0.533	0.767	3.224	1.338		
Mean ± SD	2.570 ± 0.098	0.213 ± 0.009	0.534 ± 0.015	0.797 ± 0.046	3.09± 0.125	1.311 ± 0.031		

Relative error was calculated by comparing the experimental mean value and predicted value.

**Table 3:** Box–Behnken Design (BBD) for the independent variables and corresponding response values.

Runs	Time (X1)/min	Methanol Concentration (X2)/%	Temperature (X3)/°C	Solvent-to-Material Ratio (X4)/(mL/g)	Extraction Yield/mg.g^−1^					Comprehensive Evaluation Value
					Trifolirhizin	Formononetin	Isoxanthohumol	Maackiain	Kurarinone	
1	40(0)	70(0)	70(0)	25(0)	2.117	0.172	0.432	0.574	2.428	1.040
2	40(0)	60(−1)	80(1)	25(0)	1.784	0.168	0.437	0.552	2.323	0.954
3	50(1)	80(1)	70(0)	25(0)	1.955	0.180	0.468	0.608	2.584	1.051
4	40(0)	70(0)	80(1)	30(1)	2.050	0.159	0.496	0.632	2.683	1.087
5	50(1)	70(0)	70(0)	20(−1)	1.901	0.136	0.457	0.585	2.556	1.015
6	40(0)	60(−1)	70(0)	30(1)	1.975	0.207	0.517	0.757	2.706	1.120
7	40(0)	70(0)	70(0)	25(0)	2.010	0.192	0.475	0.738	2.644	1.102
8	30(−1)	70(0)	70(0)	20(−1)	2.158	0.171	0.439	0.673	2.482	1.078
9	40(0)	70(0)	80(1)	20(−1)	2.277	0.179	0.487	0.709	2.709	1.155
10	40(0)	70(0)	60(−1)	20(−1)	2.195	0.200	0.522	0.666	2.948	1.183
11	30(−1)	70(0)	80(1)	25(0)	2.288	0.221	0.550	0.717	3.049	1.238
12	30(−1)	60(−1)	70(0)	25(0)	1.863	0.197	0.471	0.606	2.548	1.032
13	50(1)	70(0)	70(0)	30(1)	2.498	0.216	0.524	0.672	2.896	1.237
14	40(0)	70(0)	60(−1)	30(1)	1.980	0.214	0.493	0.640	2.639	1.085
15	40(0)	70(0)	70(0)	25(0)	2.036	0.198	0.500	0.624	2.748	1.107
16	40(0)	70(0)	70(0)	25(0)	2.364	0.163	0.495	0.641	2.718	1.154
17	40(0)	60(−1)	70(0)	20(−1)	1.809	0.114	0.416	0.457	2.414	0.936
18	30(−1)	80(1)	70(0)	25(0)	2.459	0.203	0.514	0.771	2.804	1.229
19	40(0)	80(1)	60(−1)	25(0)	2.489	0.208	0.522	0.782	2.926	1.260
20	50(1)	70(0)	60(−1)	25(0)	2.357	0.213	0.515	0.770	2.689	1.193
21	30(−1)	70(0)	60(−1)	25(0)	2.232	0.204	0.483	0.633	2.559	1.112
22	40(0)	60(−1)	60(−1)	25(0)	2.540	0.216	0.536	0.698	2.824	1.239
23	50(1)	70(0)	80(1)	25(0)	2.569	0.227	0.531	0.719	2.933	1.270
24	50(1)	60(−1)	70(0)	25(0)	2.212	0.204	0.488	0.627	2.539	1.104
25	30(−1)	70(0)	70(0)	30(1)	2.507	0.187	0.502	0.581	2.996	1.225
26	40(0)	80(1)	80(1)	25(0)	2.362	0.214	0.500	0.669	2.706	1.174
27	40(0)	80(1)	70(0)	20(−1)	2.266	0.151	0.443	0.637	2.441	1.077
28	40(0)	70(0)	70(0)	25(0)	2.654	0.214	0.638	0.801	1.579	1.076
29	40(0)	80(1)	70(0)	30(1)	2.490	0.177	0.476	0.695	2.597	1.170
